# Coronavirus Immunotherapeutic Consortium Database

**DOI:** 10.1093/database/baac112

**Published:** 2023-02-10

**Authors:** Jarjapu Mahita, Brendan Ha, Anais Gambiez, Sharon L Schendel, Haoyang Li, Kathryn M Hastie, S Moses Dennison, Kan Li, Natalia Kuzmina, Sivakumar Periasamy, Alexander Bukreyev, Jennifer E Munt, Mary Osei-Twum, Caroline Atyeo, James A Overton, Randi Vita, Hector Guzman-Orozco, Marcus Mendes, Mari Kojima, Peter J Halfmann, Yoshihiro Kawaoka, Galit Alter, Luc Gagnon, Ralph S Baric, Georgia D Tomaras, Tim Germann, Daniel Bedinger, Jason A Greenbaum, Erica Ollmann Saphire, Bjoern Peters

**Affiliations:** Center for Infectious Disease and Vaccine Research, La Jolla Institute for Immunology, 9420 Athena Circle, La Jolla, CA 92037, USA; Center for Infectious Disease and Vaccine Research, La Jolla Institute for Immunology, 9420 Athena Circle, La Jolla, CA 92037, USA; Center for Infectious Disease and Vaccine Research, La Jolla Institute for Immunology, 9420 Athena Circle, La Jolla, CA 92037, USA; Center for Infectious Disease and Vaccine Research, La Jolla Institute for Immunology, 9420 Athena Circle, La Jolla, CA 92037, USA; Center for Infectious Disease and Vaccine Research, La Jolla Institute for Immunology, 9420 Athena Circle, La Jolla, CA 92037, USA; Center for Infectious Disease and Vaccine Research, La Jolla Institute for Immunology, 9420 Athena Circle, La Jolla, CA 92037, USA; Center for Human Systems Immunology, Departments of Surgery, Immunology, and Molecular Genetics and Microbiology and Duke Human Vaccine Institute, Duke University, Durham, NC 27701, USA; Center for Human Systems Immunology, Departments of Surgery, Immunology, and Molecular Genetics and Microbiology and Duke Human Vaccine Institute, Duke University, Durham, NC 27701, USA; Department of Pathology, University of Texas Medical Branch at Galveston, 301 University Blvd, Galveston, TX 77555-0609, USA; Department of Microbiology and Immunology, University of Texas Medical Branch at Galveston, 301 University Blvd, Galveston, TX 77555-1019, USA; Department of Pathology, University of Texas Medical Branch at Galveston, 301 University Blvd, Galveston, TX 77555-0609, USA; Department of Microbiology and Immunology, University of Texas Medical Branch at Galveston, 301 University Blvd, Galveston, TX 77555-1019, USA; Department of Pathology, University of Texas Medical Branch at Galveston, 301 University Blvd, Galveston, TX 77555-0609, USA; Department of Microbiology and Immunology, University of Texas Medical Branch at Galveston, 301 University Blvd, Galveston, TX 77555-1019, USA; Galveston National Laboratory, University of Texas Medical Branch at Galveston, 301 University Blvd, Galveston, TX 77550, USA; Department of Epidemiology, Gillings School of Global Public Health, University of North Carolina, 135 Dauer Drive, 2101 McGavran-Greenberg Hall,CB #7435, Chapel Hill, NC 27599-7435, USA; Nexelis, a Q2 Solutions Company, 525 Boulevard Cartier Ouest, Laval, Quebec H7V 3S8, Canada; Ragon Institute of MGH, MIT and Harvard, 400 Technology Square, Cambrige, MA 02139-3583, USA; Knocean Inc., 107 Quebec Ave. Toronto, Ontario, M6P 2T3, Canada; Center for Infectious Disease and Vaccine Research, La Jolla Institute for Immunology, 9420 Athena Circle, La Jolla, CA 92037, USA; Center for Infectious Disease and Vaccine Research, La Jolla Institute for Immunology, 9420 Athena Circle, La Jolla, CA 92037, USA; Center for Infectious Disease and Vaccine Research, La Jolla Institute for Immunology, 9420 Athena Circle, La Jolla, CA 92037, USA; Center for Infectious Disease and Vaccine Research, La Jolla Institute for Immunology, 9420 Athena Circle, La Jolla, CA 92037, USA; Influenza Research Institute, Department of Pathobiological Sciences, School of Veterinary Medicine, University of Wisconsin-Madison, WI 53711, USA; Influenza Research Institute, Department of Pathobiological Sciences, School of Veterinary Medicine, University of Wisconsin-Madison, WI 53711, USA; Division of Virology, Department of Microbiology and Immunology, Institute of Medical Science, University of Tokyo, Tokyo 108-8639, Japan; The Research Center for Global Viral Diseases, National Center for Global Health and Medicine Research Institute, Tokyo 162-8655, Japan; Ragon Institute of MGH, MIT and Harvard, 400 Technology Square, Cambrige, MA 02139-3583, USA; Nexelis, a Q2 Solutions Company, 525 Boulevard Cartier Ouest, Laval, Quebec H7V 3S8, Canada; Department of Epidemiology, Gillings School of Global Public Health, University of North Carolina, 135 Dauer Drive, 2101 McGavran-Greenberg Hall,CB #7435, Chapel Hill, NC 27599-7435, USA; Department of Microbiology and Immunology, School of Medicine, 125 Marson Farm Road, Chapel Hill, NC 27599-7290, USA; Center for Human Systems Immunology, Departments of Surgery, Immunology, and Molecular Genetics and Microbiology and Duke Human Vaccine Institute, Duke University, Durham, NC 27701, USA; Carterra Inc., 825 N. 300 W.Ste, C309, Salt Lake City, UT 84103, USA; Carterra Inc., 825 N. 300 W.Ste, C309, Salt Lake City, UT 84103, USA; Center for Infectious Disease and Vaccine Research, La Jolla Institute for Immunology, 9420 Athena Circle, La Jolla, CA 92037, USA; Center for Infectious Disease and Vaccine Research, La Jolla Institute for Immunology, 9420 Athena Circle, La Jolla, CA 92037, USA; Department of Medicine, Division of Infectious Diseases and Global Public Health, University of California, 9500 Gilman Drive MC 0507, La Jolla 92093-0507, California; Center for Infectious Disease and Vaccine Research, La Jolla Institute for Immunology, 9420 Athena Circle, La Jolla, CA 92037, USA; Department of Medicine, Division of Infectious Diseases and Global Public Health, University of California, 9500 Gilman Drive MC 0507, La Jolla 92093-0507, California

## Abstract

The coronavirus disease 2019 (COVID-19) pandemic caused by the severe acute respiratory syndrome coronavirus 2 (SARS-CoV-2) has seen multiple anti-SARS-CoV-2 antibodies being generated globally. It is difficult, however, to assemble a useful compendium of these biological properties if they are derived from experimental measurements performed at different sites under different experimental conditions. The Coronavirus Immunotherapeutic Consortium (COVIC) circumvents these issues by experimentally testing blinded antibodies side by side for several functional activities. To collect these data in a consistent fashion and make it publicly available, we established the COVIC database (COVIC-DB, https://covicdb.lji.org/). This database enables systematic analysis and interpretation of this large-scale dataset by providing a comprehensive view of various features such as affinity, neutralization, *in vivo* protection and effector functions for each antibody. Interactive graphs enable direct comparisons of antibodies based on select functional properties. We demonstrate how the COVIC-DB can be utilized to examine relationships among antibody features, thereby guiding the design of therapeutic antibody cocktails.

**Database URL** https://covicdb.lji.org/

## Introduction

The coronavirus disease 2019 (COVID-19) pandemic, caused by the emergence of the severe acute respiratory syndrome coronavirus 2 (SARS-CoV-2) and associated variants, spurred the development of a wide range of antibody therapeutics by groups in academic and industry settings. Choosing candidate therapeutic antibodies, or combinations thereof, from this large pool to generate an effective and potent therapeutic antibody treatment relies on uniform assessment of the biological activities of these antibodies. However, the experimental assays and conditions under which these therapeutic antibodies have been evaluated and characterized differ among laboratories. Thus, making comparisons among antibodies based solely on laboratory-specific experimental measures is challenging. To overcome this challenge, the Coronavirus Immunotherapeutic Consortium (COVIC) (1) was created to standardize the evaluation of therapeutic antibodies and enable meaningful comparisons and analyses. To date, the COVIC consortium compiled a panel of nearly 400 antibodies against the SARS-CoV-2 spike protein that were contributed by 60 different groups. Eight different reference laboratories (RLs) tested these antibodies side by side in various assays to assess different functional activities such as neutralization potency, binding affinity, *in vivo* protection, effector functions and pharmacokinetics. At the time of antibody submission, antibody identities were blinded through the assignment of code names to address concerns regarding intellectual property rights for antibody contributors (ACs) during a time in the pandemic when the field was highly competitive. Recently, we asked the ACs if they were willing to unblind the antibody identities. Forty percent of ACs gave explicit permissions to unblind while the rest of the antibody identities continue to remain blinded.

The approach taken by the COVIC builds on the previous experience of the Viral Hemorrhagic Fever Immunotherapeutic Consortium (VIC) (2). The objective of the VIC was the rapid discovery and evaluation of therapeutic antibodies against the Ebola virus surface glycoprotein. Upon initiating the COVIC project, the VIC leadership was asked what adjustments they would recommend based on their previous experience. One recommendation was to ensure that data management, including capture, harmonization and access, adhered to FAIR (Findability, Accessibility, Interoperability, Reuse) principles (3), so the data could be more easily repurposed in the future. In the VIC project, data were handled with spreadsheets, which had the advantage of requiring no setup time during a crisis, but did not facilitate field-wide usage of a more permanent or interactive database essential for a broader crisis such as the SARS-CoV-2 pandemic.

To handle data generated by the COVIC consortium, we designed the COVIC database (COVIC-DB). The COVIC-DB can be considered as an experimental database as the data that it contains are generated by several experimental assays carried out by different partner laboratories. The objective of the COVIC-DB is to address three main goals: (i) to ensure that ‘data submissions’ adhere to agreed-upon standards; (ii) to enable blinded ‘access’ to data about antibody characteristics for the broader community, while allowing contributors to see how their specific antibodies performed and (iii) to provide ‘data analysis’ tools for the aggregate data. The key differences between the VIC and COVIC-DB in terms of data handling are that the COVIC-DB has a validation system put in place to perform automated quality control (QC) before accepting antibody submissions. The validation system also ensures that the terms in the submitted data fall within the controlled vocabulary so that the data reflect the general understanding of the antibodies and assays. The VIC did not have a dedicated website displaying all the Ebola antibody data that it generated. COVIC-DB fulfills that purpose for the SARS-CoV-2 antibody data. This study thus describes how these three goals were achieved by the COVIC-DB and demonstrates how the database can be used to gain insights into the selection of antibodies or antibody cocktails for clinical applications.

## Methods

### Database implementation

The database utilizes a PostgreSQL relational database management system with the application backend implemented as a Django web framework and the frontend/user interface built using D3.js and Angular. The 3D viewer tool for visualizing the epitope footprints on Spike is implemented using iCn3D, a structure viewer written in JavaScript (4).

### Data validation

Controlled terminology from open community ontologies and validation rules are collaboratively edited using a Google Sheet, and a git repository stores the versioned terminology tables and validation code. A Python library and command-line tool configure new submission templates, generate empty Excel files for users, then read and validate filled Excel files. If a validation error occurs, the submitter receives a copy of the Excel file with problem cells highlighted and annotated with a description of the problem and suggested resolution. If there are no validation errors, submitted data are converted to tab-separated values (TSV) format and stored in an internal git repository behind the La Jolla Institute for Immunology (LJI) firewall that preserves a version history of all submitted data. Confidential data such as antibody names are used as internal reference and are not stored on any public facing systems. Configuration files and validated TSV files are then read into the database by assigned blinded identifiers.

### Tutorial and documentation

We provided a tutorial page on the website that explains how to navigate through the database website. In addition, a ‘Documentation’ page lists the definitions of the scientific terms in the website. The pages can be accessed by clicking on ‘Tutorial’ and ‘Documentation’, respectively, on the left-most panel of the dashboard.

## Results

### COVIC-DB design following FAIR principles

COVIC-DB was designed from the outset to ensure that the data are FAIR. ‘Findability’ was established by assigning to the data globally unique and persistent identifiers upon submission. The primary identifiers here are the COVIC-IDs that are uniquely assigned to each antibody (e.g. COVIC-1, COVIC-2, etc.). We will further publicize the database in relevant indexes as the first stable release is completed. ‘Accessibility’ is achieved through the open access to the COVIC-DB dataset from https://covicdb.lji.org/. The data are available as an interactive Hyper Text Markup Language (HTML) table and may also be downloaded in a comma-separated values (CSV) format for computational purposes. For ‘interoperability’, all vocabularies in the data are sourced from open community ontologies, such as National Center for Biotechnology Information (NCBI) Taxonomy and Protein Ontology to describe an antibody’s host or isotype. For the experimental data that are hosted, we have ensured that each assay type is formally described using identifiers from the Ontology for Biomedical Investigations (OBI) (5), the Ontology for Immune Epitopes (6) and other open community ontologies. As part of this process, multiple new term requests were made as part of new assay types captured in OBI. These new terms are now part of OBI and will provide the broader community a more detailed vocabulary to describe such experiments in general. To be ‘reusable’, all data in the database are released without license restrictions.

### Data submission and validation

To ensure broad participation in the COVIC and enable evaluation of as many antibodies as possible, the intellectual property rights of ACs, who were working in laboratories located in academic, government and independent research institutions, as well as those as in industry, must be protected. To this end, COVIC headquarters at LJI served as an intermediary between the ACs and RLs to manage antibody shipments and the blinding process. ACs uploaded basic information about each antibody into the COVIC-DB, including the name by which the AC referenced the antibody termed ‘Antibody Name’ (note that ACs could select any name that they wished, to add an additional degree of blinding), ‘Host’ (the organism from which the entry was isolated) ‘Isotype’, ‘Light chain’, ‘Heavy chain’, ‘Antibody details’ and ‘Structural data’ (to allow ACs to specify whether they wanted structural analyses) and ‘Antibody comment’. These fields were validated against controlled vocabulary drawn from the NCBI Taxonomy (7) and the Protein Ontology (8). The only required fields on the antibody submission form were ‘Antibody Name’ and ‘Host’. ACs were encouraged to reference any data they might have already collected or other information that would be salient for the COVIC analyses. Upon submission, the antibodies were assigned numeric COVIC-IDs, and the linkage between ID and antibody was made available only to the contributor of the specific antibody, the COVIC program manager and the program officers of the two main funding bodies [Bill & Melinda Gates Foundation (BMGF) and National Institute of Allergy and Infectious Diseases]. The ACs received individual login information that allowed them to access an account to follow the progress of the analyses. Antibody aliquots labeled with COVIC-IDs were prepared and then sent from LJI to the RLs for testing.

**Figure 1.**
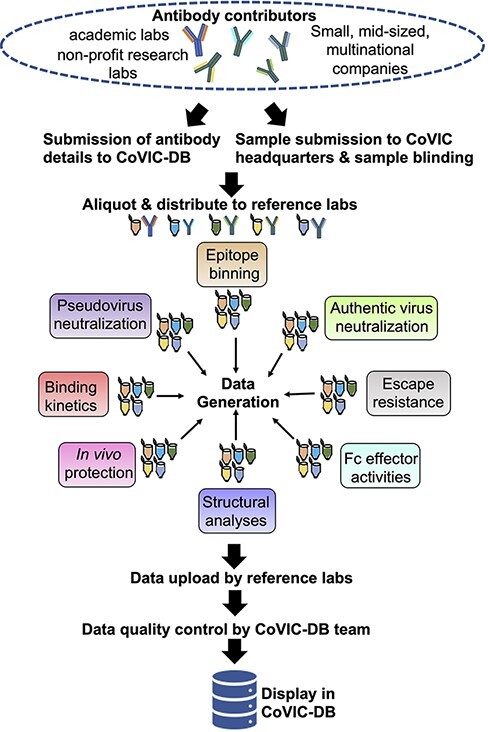
Illustration of the workflow, beginning from antibody collection, to data generation and deposition in the COVIC-DB.

Each RL measured a specific functional property of the antibodies in the COVIC panel. These functional properties included: affinity for the full-length ectodomain of SARS-CoV-2 spike that included residues 1–1208 with aspartic acid (D) at position D614 (D614; also commonly referred to as the Wuhan (9, 10) and WA-1 strains (11)), the D614G variant (12), the B.1.351 variant that was first identified in South Africa (13) (hereafter referred to as Beta variant), the recent B.1.1.529 (Omicron) variant (14, 15) as well as for the soluble receptor-binding domain (RBD; residues 318–591) and N-terminal domain (NTD; residues 14–305), *in vitro* neutralization of infection by pseudotyped (D614, Beta, B.1.617.2 (16) which was first identified in India and is commonly known as the Delta variant), or authentic virus (D614 and Beta); *in vivo* protection in a mouse model of SARS-CoV-2 infection (SARS-CoV-2/human/USA/WA-CDC-WA-1 (GenBank MN985325) (17), high resolution epitope binning, negative stain electron microscopy (on select antibodies) (1), antibody blocking of angiotensin-converting enzyme 2 (ACE2) receptor binding to RBD, mouse blood serum antibody concentration determined from pharmacokinetic assays, antibody escape mutations, Fc effector functions against the SARS-CoV-2 displayed on beads, and affinity for a battery of Fc receptors (FcRs). The data types generated and workflow are summarized in Figure 1. The resultant data generated from these experiments were uploaded by the RLs to the COVIC-DB through a login-protected submission portal using standardized spreadsheet submission templates in which each of the antibody COVIC-IDs was linked to experimental characteristics.

For each of the assays performed, a data submission template was generated by the COVIC-DB team in consultation with the RLs performing the assay. Once the RLs uploaded a completed submission template via the COVIC-DB submission portal, the files were automatically checked for consistency with the agreed-upon format. Validation checks included matching antibody IDs against the list of COVIC-IDs and control antibodies, ensuring that required fields were filled and ‘Not Applicable (NA)’ fields were correctly entered, checking numeric formats and syntax for limits of measurement thresholds, checking ranges for scores (numeric and categorical) and enforcing standardized syntax for specifying mutations. Errors were reported back to the submitters in real time as lists of validation messages that corresponded to relevant cells in the uploaded template. Only validated submissions were accepted. The submission system was also version-controlled to enable the RLs to upload corrected data. After undergoing appropriate quality checks, the processed data were then uploaded to the main COVIC-DB site for public viewing. Most validation issues occurred for the initial submissions by the RLs, and the frequency of these issues decreased over time. The most common validation issues encountered were inconsistencies in terminology, wherein terms did not match the controlled vocabularies and references were made to invalid antibody IDs, which were typically associated with control antibodies rather than COVIC-IDs and often reflected misunderstanding of the submission format. Identifying such inconsistencies early rather than at the time of data analysis simplified correction and ensured that the data package generated was consistent.

### Data access through the COVIC-DB dashboard

**Figure 2.**
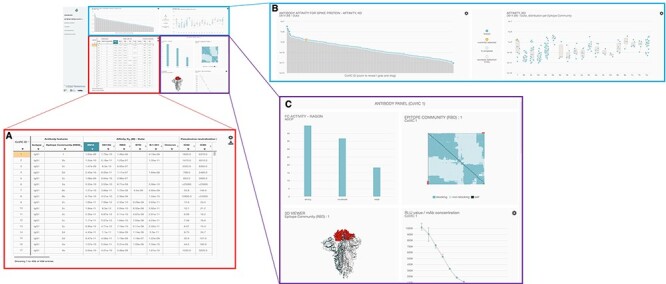
Overall layout of the COVIC-DB landing page. (A) contains the summary table showing results for different assays carried out for COVIC antibodies. The user has highlighted the upper left-most cell in orange by clicking on the cell. (B) displays graphical representations of the affinity data shown in the summary table. (C) highlights the ‘Antibody Panel section of the dashboard that appears when the user selects an individual antibody (here, COVIC-1).

All data submitted data that passed QC checks were made publicly accessible through the COVIC-DB dashboard (https://covicdb.lji.org/), from which they can be freely downloaded and analyzed. The CoVIC-DB dashboard contains multiple panels (Figure 2). The summary table (Figure 2A) displays each CoVIC antibody in an individual row with columns corresponding to features or experimental measurements of the antibody. The columns display (i) the unique ‘CoVIC-ID’, (ii) the antibody ‘Isotype’, (iii) the ‘Epitope Community (RBD)’ assigned based on results of high-resolution epitope binning that examined the ability of antibodies to compete with one another for binding to soluble receptor-binding domain (RBD) and (iv) ‘Affinity – Duke’ lists binding affinities of the antibody to various spike constructs expressed as molar *K*_D_ values (dissociation constant). These affinity values were measured by the RL at Duke and were determined for full-length ectodomain of Spike protein for ‘*D614*’, ‘*D614G*’, the Beta variant of which is represented by the ‘*B.1.351*’ sub-column, and ‘*Omicron*’. Meanwhile, ‘RBD’ and ‘NTD’ sub-columns list *K*_D_ values for RBD and NTD, respectively. All columns have a ‘Filter and Sort’ feature to organize the content according to parameters defined by the user or to highlight particular antibodies. The full list of columns captured in the dashboard and their definitions are shown in Table 1.

**Table 1.** Descriptions of columns and sub-columns present in the COVIC-DB dashboard table

Column name	Sub-column name	Description
COVIC-ID		The COVIC label for the antibody
Antibody features	Isotype	The antibody isotype
	Epitope Community (RBD)	The epitope community to which the antibody belongs, determined using epitope binning in a classical sandwich assay format by Carterra
Affinity, *K*_D_ (M), Duke	D614	Binding affinity of antibody to full-length Spike (D614; aa 1-1208)
	D614G	Binding affinity of antibody to full-length Spike (D614G; aa 1-1208)
	RBD	Binding affinity of antibody to RBD of D614 (aa 318-591)
	NTD	Binding affinity of antibody to NTD of D614 (aa 14-305)
	B.1.351	Binding affinity of antibody to full-length Spike B.1.351 (Beta) variant (aa 1-1208)
	Omicron	Binding affinity of antibody to full-length Spike (Omicron) variant (aa 1-1208)
Pseudovirus neutralization; D614, Beta, Delta (ng/ml)	IC_50_	IC—concentration of antibody needed to neutralize 50% of infection by pseudovirus bearing the indicated SARS-CoV-2 spike protein relative to infection in the absence of antibody, measured by Nexelis
	IC_80_	Concentration of antibody needed to neutralize 80% of infection by pseudovirus bearing the indicated SARS-CoV-2 spike protein relative to infection in the absence of antibody, measured by Nexelis
	IC_90_	Concentration of antibody needed to neutralize 90% of infection by pseudovirus bearing the indicated SARS-CoV-2 spike protein relative to infection in the absence of antibody, measured by Nexelis
Authentic virus neutralization (ng/ml)—UNC	IC_50_ (WA-1)	Concentration of antibody needed to neutralize 50% of infection by authentic wild-type SARS-CoV-2 (D614, strain WA-1) measured at the University of North Carolina
	IC_80_ (WA-1)	Concentration of antibody needed to neutralize 80% of infection by authentic wild-type SARS-CoV-2 (D614, strain WA-1) measured at the University of North Carolina
	IC_50_ (B.1.351)	Concentration of antibody needed to neutralize 50% of infection by authentic SARS-CoV-2 Beta variant (B.1.351), measured at the University of North Carolina
	IC_80_ (B.1.351)	Concentration of antibody needed to neutralize the Beta variant of SARS-CoV-2 (B.1.351) authentic virus to 50% of its original concentration, measured by UNC
Authentic virus neutralization (ng/ml)—UTMB	IC_50_	Concentration of antibody needed to neutralize 50% of infection by authentic wild-type SARS-CoV-2 (D614, strain WA-1), measured at the University of Texas Medical Branch at Galveston
	IC_80_	Concentration of antibody needed to neutralize 80% of infection by authentic wild-type SARS-CoV-2 (D614, strain WA-1), measured at the University of Texas Medical Branch at Galveston
	IC_90_	Concentration of antibody needed to neutralize 90% of infection by authentic wild-type SARS-CoV-2 (D614, strain WA-1), measured at the University of Texas Medical Branch at Galveston
*In vivo* protection survival (%)	K18 mice	Ability of the antibody to protect mice infected with SARS-CoV-2. Percentage of K18 hACE2 transgenic mice surviving at 10 days post-infection, measured at Texas Biomedical Research Institute
Fc activity—Ragon	ADCP	The qualitative value of antibody-dependent cellular phagocytosis (ADCP), an assay that measures the antibody-initiated phagocytosis of phagocytic cells by binding its Fc domain to a specific receptor on monocytes using a phagocytic score
	mADCP	*‘mADCP*’ refers to the qualitative value of phagocytic score of antibody-dependent cellular phagocytosis in which the specific receptor is present on a mouse cell
	ADNP	The qualitative value of antibody-dependent neutrophil phagocytosis (ADNP), an assay that measures the antibody-initiated phagocytosis of neutrophils by binding its Fc domain to a specific receptor on neutrophils using a phagocytic score
	mADNP	*‘mADNP*’ refers to the qualitative value of antibody-dependent neutrophil phagocytosis, an assay that measures the antibody-initiated phagocytosis of neutrophils by binding its Fc domain to a specific receptor on mouse neutrophils using a phagocytic score
	ADCD (median fluorescence intensity (MFI C3)	Qualitative value of antibody-dependent complement deposition, an assay to measure complement component C3b on the surface of target cells using MFI
	ADNKA % CD107a+	Qualitative value of assay measuring the percent of natural killer (NK) cells expressing CD107a as a result of antibody binding
	ADNKA % MIP-1b+	Qualitative value of assay measuring the percent of NK cells expressing MIP-1b as a result of antibody binding
	FcgR2aR (MFI)	Assay measuring antibody affinity for FcgR2aR receptor on human cells using MFI
	FcgR3aV (MFI)	Assay measuring antibody affinity for FcgR3aV receptor on human cells using MFI
	FcgR2b (MFI)	Assay measuring antibody affinity for FcgR32b receptor on human cells using MFI
	FcgR3b (MFI)	Assay measuring antibody affinity for FcgR3b receptor on human cells using MFI
	FcRn (MFI)	Assay measuring antibody affinity for FcRn receptor on human cells using MFI
	mFcR2 (MFI)	Assay measuring antibody affinity for FcR2 receptor on mouse cells using MFI
	mFcR3 (MFI)	Assay measuring antibody affinity for FcR3 receptor on mouse cells using MFI
	mFcR4 (MFI)	Assay measuring antibody affinity for FcR4 receptor on mouse cells using MFI
Complex formation inhibition (%)	Spike:ACE2	Defines the extent to which an antibody inhibits the formation of a complex between the Spike protein and ACE2 receptor (100% denotes total inhibition)
UTMB—antibody-dependent monocyte phagocytosis (ADMP)	Fold increase over virus control	The ratio of mean phagocytosis, an assay measuring the mean percentage of infected cells undergoing phagocytosis, for an antibody over the no-antibody control
Sequence/structural features	Escape	
Pharmacokinetics (PK)	GMC for pre-Spike immunoglobulin G (IgG) ELISA (ELU/ml)	An enzyme-linked immunosorbent assay (ELISA) measuring geometric mean concentration (GMC) of an IgG antibody against pre-Sspike in ELISA units per milliliter (ELU/ml)
	GMC for RBD IgG ELISA (ELU/ml)	An ELISA measuring geometric mean concentration of an IgG antibody against RBD in ELU/ml

The top-most dashboard panel (Figure 2B) displays a graphical representation of data from a specific table column. The user can select the property to be displayed by clicking on a column header in the summary table (Figure 2A). For example, clicking on the D614 column sub-header under ‘Affinity’ produces a plot of the binding affinities of each antibody for the full-length ectodomain of the spike D614 variant corresponding to the COVIC-ID arrayed on the *x*-axis (Figure 2B, left). Clicking on the gear icon in the top right corner of the graph allows the user to enlarge the plot, switch between linear and log scales and zoom in on a section of the graph. A boxplot of the same affinity information, grouped by either competition bin or antibody isotype, is also displayed (Figure 2B, right). The display options are customizable, and the plots can be downloaded as .png files.

The third dashboard panel, also referred to as the ‘antibody panel’ (Figure 2C), summarizes data for a specific antibody. Individual antibodies can be selected either by clicking on a row in the summary to highlight it or by using the filter tool (Figure 2A). The view in the antibody panel includes the following: (i) FcR-binding affinities, immune effector functions and phagocytosis distribution, (ii) epitope community, (iii) visualization of epitope community on the 3D structure of Spike (also see the 3D visualization of epitope communities, mutations, and immunogenicity on Spike section), (iv) *in vivo* protection and weight loss in mice post-infection and (v) Relative luminescence units (RLU) vs. monoclonal antibody (mAb) concentration for the Nexelis pseudovirus neutralization assay. A more detailed view of the data for a given antibody is available by clicking on the ‘Antibody Details’ header that links to a page listing more comprehensive results of each assay carried out for the antibody (Supplementary Figure S1). When applicable, analysis of negative stain electron microscopy data is also included on this page.

### 3D visualization of epitope communities, mutations and immunogenicity on Spike

Given the rapidly changing landscape of the SARS-CoV-2 virus and the frequent emergence of variants of concern (VOCs), the ability to identify which mutations in the VOCs are likely to impact antigen-binding properties of a specific antibody is a desired feature. To provide a visual interpretation template, we incorporated a 3D visualization tool, iCn3D (4), in the main page of the database website. By clicking on a COVIC-ID in the summary table (Figure 2C), the color-coded epitope footprint, as defined from negative stain electron microscopy studies (1) or predicted from high-resolution epitope binning corresponding to the highlighted antibody epitope is displayed on the structure of the Spike protein (Supplementary Figure S2). Clicking on the panel displaying the 3D structure reveals a new page that lists VOCs of interest and allows close inspection of where mutations within the VOCs occur as well as their proximity to the epitope communities. In addition to the mutations in the VOCs, escape mutations identified for some candidate therapeutic antibodies can also be viewed by selecting from another dropdown menu. Regions on the Spike protein have different levels of immunogenicity. To visualize these regions that have high and low immunogenicity on the 3D structure of Spike, we used data from over 250 published antibody epitope mapping studies curated in the Immune Epitope Database (IEDB) (18). This set of antibodies in the IEDB used to assess the immunogenicity is distinct from the set of antibodies present in the COVIC-DB. The data are summarized by calculating response frequency values for each position in the Spike protein by assessing the epitopes overlapping the position and the number of individuals that showed an immune response to these epitopes vs. those that did not, as implemented in the IEDB ImmunomeBrowser tool (19). A button activates display of these data, which color the Spike positions in colors ranging from blue, to white to red that represent low, moderate and high immunogenicity, respectively.

### Analysis of data in COVIC-DB can reveal relationships among different types of measured antibody data

We leveraged the information deposited into the COVIC-DB to gain a better understanding of how data gathered by different assays were correlated using Spearman’s correlation coefficient in a pairwise fashion.

#### Neutralization of pseudovirus predicts neutralization of authentic virus

**Figure 3.**
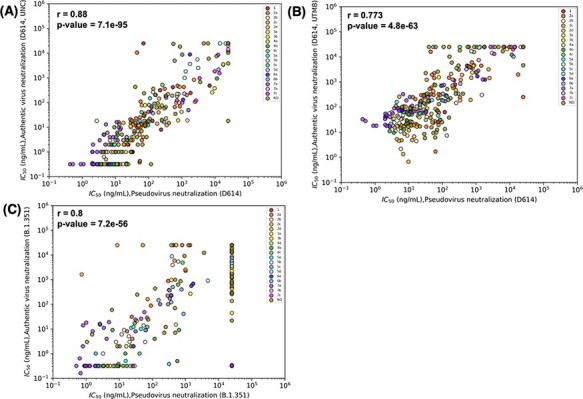
Results for pseudovirus and authentic virus neutralization assays strongly correlate. Correlations were estimated by Spearman’s correlation coefficients and relate neutralization of pseudovirus bearing D614 and (A) authentic D614 virus (measured at University of North Carolina, Chapel Hill (UNC)), (B) D614 (measured at University of Texas Medical Branch at Galveston (UTMB)) and (C) authentic Beta (B.1.351) virus (measured at UNC). Data points are colored according to the epitope community to which the antibody belongs. ND: Epitope community not determined.

We first examined the association among values for half-maximal inhibitor concentration (IC_50_) obtained for neutralization of infection by pseudovirus or authentic virus with either D614 SARS-CoV-2 Spike or the Beta variant. Pseudovirus IC_50_ values were strongly correlated with authentic virus neutralization in both cases (*r* = 0.88 for D614, *r* = 0.8 for Beta) (Figure 3), indicating that pseudovirus neutralization activity of an antibody is a good predictor of its ability to neutralize authentic virus in these assays. This finding is particularly important as pseudovirus neutralization can be performed under less stringent biosafety containment conditions (biosafety level (BSL)-2 vs BSL-3) that require fewer resources, potentially allowing for analysis of a broader panel of antibodies. However, for some antibodies (e.g. COVIC-271), the results for authentic and pseudovirus neutralization assays were discordant. This finding may have biological importance and could guide decisions about whether antibodies in certain epitope communities or that have particular features should be analyzed using both assays. Overall, the COVIC-DB data show that pseudovirus neutralization is an efficient tool for screening antibodies for their neutralizing capacity.

#### Epitope community and binding affinity for Spike are related to neutralization potency

**Figure 4.**
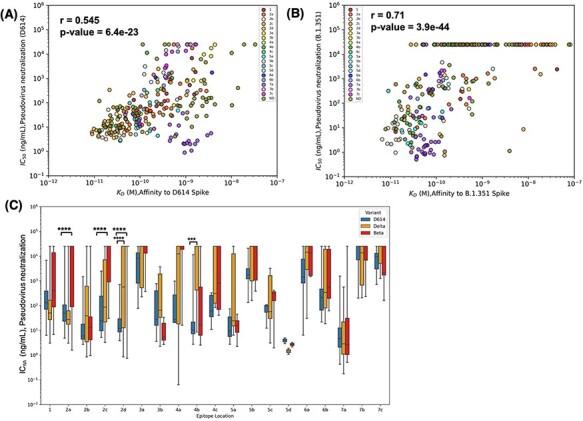
Epitope location and binding affinity together drive neutralization. (A) Spearman’s correlation between binding affinity and neutralization of pseudovirus bearing D614 spike. Data points are colored according to the epitope community to which the antibody belongs. ND: Epitope community not determined. (B) Correlation between binding affinity and neutralization of pseudovirus bearing Beta spike. (C) Epitope-wise comparison of pseudovirus neutralization of wild-type SARS-CoV-2 (D614, blue bars) and its variants Beta (red bars) and Delta (orange bars). Statistical significances were calculated using Welch’s *t*-test (****P < 0.0001,*** *P* < 0.001, **P < 0.01, *P < 0.05). Antibodies are grouped according to the epitope community, and the bars represent the mean binding affinity (note that Community 5d includes only two antibodies).

The binding affinity of an antibody for Spike is expected to be tightly correlated with its neutralization potency. However, binding affinity showed only a moderate positive correlation with pseudovirus IC_50_ values (*r* = 0.545) for D614 and somewhat higher (*r* = 0.71) affinity for Beta (Figure 4A, B), suggesting that other factors could play a role in neutralization potency. We thus investigated the role of Spike epitope, as assessed by determining relationships between epitope community and neutralization potency and how overlapping mutations in Beta and Delta variants impacted neutralization (Figure 4C). The epitope communities were defined based on competitive binding assays and negative stain electron microscopy (1). Communities RBD-1 and RBD-2 include the receptor-binding motif (RBM). RBD-3 was also initially predicted to include the RBM (1), but finer classification with additional antibodies that arrived later suggested that RBD-3 lies outside of the RBM. Communities RBD-4 through RBD-7 lie outside of the RBM as well. RBD-4 and RBD-5 comprise the outer face of the RBD, while RBD-3, RBD-6 and RBD-7 include the inner face of the RBD. We observed antibodies in the RBD-5a, and RBD-7a epitope communities have high pseudovirus neutralization against D614, followed by antibodies belonging to communities RBD-2b and RBD-4b (Figure 4C). RBD-1 antibodies show increased pseudovirus neutralization against Delta, but have varying activity against Beta. Meanwhile, RBD-2 antibodies show a decrease in pseudovirus neutralization against Beta, but antibodies within the RBD-2 sub-community RBD-2a showed no increase or decrease in pseudovirus neutralization of Delta. Relative to D614, RBD-3b antibodies have improved pseudovirus neutralization against Beta, but not against Delta. Antibodies in the RBD-4a community had decreased pseudovirus neutralization against both Beta and Delta relative to D614. RBD-7a antibodies show consistent pseudovirus neutralization against D614, Beta and Delta. Thus, these results demonstrate that a combination of antibody binding affinity and epitope location is vital as together they can explain much of the observed neutralization potency.

#### Mutations in the Beta variant alter antibody binding to spike in an epitope-specific manner

**Figure 5.**
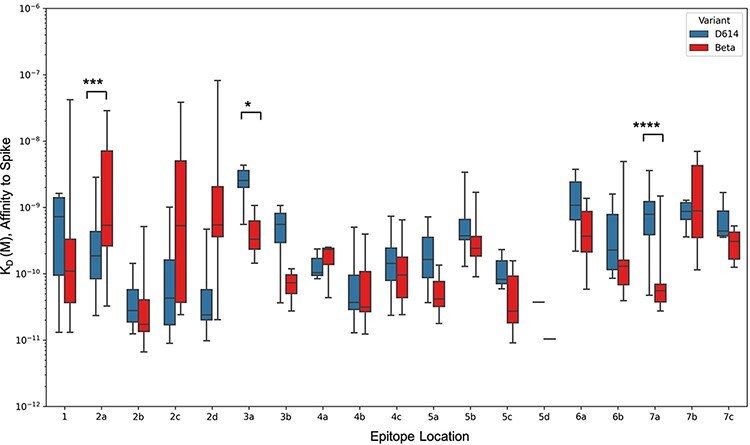
Epitope-wise comparison of antibody binding affinity for Spike D614 (blue bars) and Beta (red bars). Statistical significances were calculated using Welch’s *t*-test (****P < 0.0001,*** *P* < 0.001, **P < 0.01, *P < 0.05). Antibodies are grouped according to the epitope community, and the bars represent the mean binding affinity (note that Community 5d includes only two antibodies).

Mutations in earlier and current VOCs can drastically affect binding of some antibodies (20–22). Overall, we observed a weak–moderate positive correlation (*r* = 0.37) in binding affinities of D614 vs. Beta (Supplementary Figure S3). To gauge how affinity is affected by VOC mutations in terms of epitope location, we assessed the binding affinity of antibodies belonging to each epitope community for both the D614 and Beta variant (Figure 5). Antibodies belonging to subcommunities 2a, 2c and 2d showed a decrease in binding affinity for Beta relative to D614. However, antibodies belonging to RBD-3, as well as those in subcommunities RBD-5a, RBD-5b, RBD-6a, RBD-6b and RBD-7a showed increased binding affinity to the Beta variant.

## Discussion

COVIC-DB catalogs the functional properties as measured under identical assay conditions of over 400 antibodies and antibody-like molecules obtained from different sources. In addition to the antibody data, the database and associated website provide tools for directly analyzing the data, thereby assisting the user in carrying out rapid and simple comparisons without having to download the data. The COVIC-DB thus provides a platform for users to identify trends in the aggregated data that reveal which antibody features are important for neutralization of D164 as well as the Beta and Delta VOCs. Using the COVIC-DB framework, we could discern that pseudovirus neutralization was generally a good predictor of authentic virus neutralization. We also found that the location of the epitope targeted by antibody in conjunction with affinity is an important predictor of neutralization potency. Contrasting the neutralization potency between the D614 and Beta or Delta variants by epitope community revealed which epitope locations are affected by which mutations in the VOCs. For example, the RBD-2a antibody COVIC-49 has pseudovirus neutralization similar IC_50_ values of 57.4 ng/ml and 35.7 ng/ml against D614 and Delta, respectively, but decreased neutralization (IC_50_ = 2540 ng/ml) against Beta. Another RBD-2a antibody, COVIC-52, showed a similar trend [IC_50_ = 221 ng/ml (D614), 134 ng/ml (Delta), no neutralization of Beta]. On the other hand, the RBD-4b antibody COVIC-268 has similar IC_50_ values against D614 and Beta (9.28 ng/ml and 4.8 ng/ml, respectively) but does not neutralize Delta (IC_50_ > 25 000 ng/ml). Examples of antibodies that show strong pseudovirus neutralization and are unaffected by mutations in Beta or Delta include COVIC-59, COVIC-122, and COVIC-312, which are all assigned to RBD-7a. Obtaining this type of information was made possible only by consolidating the large-scale efforts and partnerships of different laboratories into a single repository.

Multiple resources that contain information about SARS-CoV-2-specific antibodies or provide details of literature studies describing these antibodies have been established (22). These include the Coronavirus Antibody Database (23), which has been compiling antibody information from the literature and patents since the beginning of the pandemic, and the Coronavirus Antiviral Research Database (24) that has a broader scope and also includes small molecule data. Antibody epitope recognition data are also captured through literature curation in the IEDB. The main distinguishing factor of COVIC-DB compared to these and other resources is that (i) it includes data on antibodies not published elsewhere and directly compares nearly all therapeutic candidates and (ii) the measured data are generated in a centralized fashion that facilitates direct quantitative comparisons.

Several studies on specific anti-SARS-CoV-2 antibodies have provided detailed information and offer new insights that can be used for antibody optimization (25, 26, 27). For example, these studies investigated the neutralization ability of plasma obtained from volunteers who were either vaccinated or recovered from infection with the different SARS-CoV-2 variants. Further characterization included isolation and sequencing of single memory B cells to analyze the sequences of SARS-CoV-2-binding antibodies and evaluate their binding ability to the RBD of different variants.

While the COVIC-DB does not provide such in-depth information about each antibody like these studies, the database provides different information that highlights the range of achievable experimental results. The breadth of functional activities surveyed for each antibody affords users the opportunity to examine relationships among different antibody properties. Although the results of our analyses recapitulate those of earlier reports, the focus of this paper is on the novelty of our approach to providing a framework for data collection, representation and sharing of deidentified antibodies that allow ACs to determine how their antibodies fare in the context of a broader field.

The FAIR design of COVIC-DB makes cross-linking the data with other databases straightforward. Although to date, the work has focused on implementing the COVIC-DB and uploading content, on the backend, we have begun to integrate the content with information from the IEDB, which broadly tracks the molecular targets of immune responses in SARS-CoV-2, for calculating the response frequencies, which are a measure of how immunogenic each region on the SARS-CoV-2 Spike protein is. Additionally, we are connecting with initiatives tracking viral variants such as the Accelerating COVID-19 Therapeutic Interventions and Vaccines/Tracking Resistance and Coronavirus Evolution initiative.

The overall goal of rapidly evaluating therapeutic agents produced by commercial and academic researchers in a blinded fashion in partnership with RLs that generate comparable, quantitative data has broad applicability. The biggest challenges we encountered as part of the COVIC-DB project was establishing data submission standards, including anticipating outlier cases and communicating and enforcing these standards with all participants. During the height of the pandemic in particular, having the full attention of laboratories that were working at capacity on multiple projects was challenging. Such delays could be avoided (or at least reduced) if the infrastructure for projects like COVIC-DB would be maintained outside of such intense periods to provide a framework for data management in a ‘ready-to-deploy’ mode that can be adopted to new targets and challenges.

Entities involved in antibody manufacturing efforts, especially during the antibody development stage, will benefit immensely from the COVIC-DB as our data provide benchmarks on what can be achieved. Another advantage of the COVIC-DB design is that it serves as a basis for the funders of COVIC to select antibodies for further development by providing consistently generated data that can be directly compared. We hope that reporting our experience with this database development will serve as a useful reference for similar future efforts and this, in turn, makes the COVIC-DB a valuable contribution to the field.

## Supplementary Material

baac112_Supp

## Data Availability

The data presented here can be freely downloaded from the COVIC-DB database (https://covicdb.lji.org/). Links to the various assay types described here can also be downloaded from https://covicdb.lji.org/downloads.
