# Supplementary material for: Coronavirus Immunotherapeutic Consortium Database
Source: Database (Oxford). 2023 Feb 10;2023:baac112. doi: 10.1093/database/baac112 (PMC9913043; doi:10.1093/database/baac112)
Supplement: baac112_Supp [file baac112_supp.zip › suppl_data/Revised_Supp_Material.doc]

**Supplementary Material for**

# “Coronavirus Immunotherapeutic Consortium Database (CoVIC-DB)”

**Supp. Figure 1:** Snapshot of the antibody details page obtained by clicking on the row for CoVIC-1 in the dashboard summary table, with ‘Antibody Details’ shown on the left side of the dashboard. The page provides a summary of the results of all assays carried out COVIC-1. Some examples include effector functions (**A**), pseudovirus neutralization (**B**), and authentic virus neutralization (**C**).

**Supp. Figure 2:** Visualization of residues present in the epitope community for COVIC-1, which is a member of the RBD-1 community. PDB 7DDD was used for mapping the residues of the epitope community.

**Supp. Figure 3**: Spearman’s correlation of binding affinities for D614 Spike and Beta Spike. Data are points are colored according to epitope community to which the antibody belongs. ND: Epitope community not determined.
